# Use of Colorimetry for the Measurement of Intradermally Injected Histamine-Induced Erythema in Healthy Dogs: A Proof-of-Concept Study

**DOI:** 10.3390/vetsci12060590

**Published:** 2025-06-15

**Authors:** Ana Petak, Elisa Samuel (Badulescu), Svetlina Aleksandrova, Evi I. Sofou, Manolis K. Chatzis, Manolis N. Saridomichelakis

**Affiliations:** Clinic of Medicine, Faculty of Veterinary Science, University of Thessaly, Trikalon Str. 224, 43 132 Karditsa, Greece; ana.petak@gmail.com (A.P.); mchatzis@vet.uth.gr (M.K.C.)

**Keywords:** colorimetry, erythema, flare reaction, histamine, intradermal test, wheal

## Abstract

The interpretation of intradermal test results may improve if wheal erythema is measured objectively using colorimetry. Our aim was to find the necessary number of erythema colorimetric measurements, taken before and after intradermal injections of histamine 0.01% (H1), histamine 0.001% (H2), histamine 0.0001% (H3) and a negative control (NC). We also aimed to assess whether erythema should be measured at the wheal center or border, and to investigate whether erythema appears beyond histamine wheals (flare), as observed in humans. Duplicate injections of the four solutions were administered on the lateral thorax of seven healthy dogs and erythema was measured by removing and repositioning the colorimeter probe. Colorimetry can differentiate the change in erythema (*Δ*E) at the center (*Δ*Ec), but not at the border, of the H1 wheals from the *Δ*E of NC and H3 wheals. Reliable ways to calculate *Δ*Ec were to use the first positive a* axis value of the colorimeter before and after the injection or to obtain the first seven positive a* axis values, excluding the highest and lowest, and calculated the mean of the remaining five. Unlike in humans, intradermal injection of histamine, even at a concentration of 0.01%, did not induce a flare reaction in healthy dogs.

## Simple Summary

An intradermal test is commonly performed in dogs with allergies to determine whether they are sensitized to various environmental allergens. Unfortunately, the interpretation of test results is subjective and not highly reproducible. We investigated whether this could be improved by using a colorimeter, a device that measures the redness of the skin. The colorimeter was used to measure the redness of wheals that were induced in seven healthy dogs by intradermal injections of strong, intermediate and weak solutions of histamine and saline. Our results showed that colorimetric measurements detected a greater increase in skin redness at the center of the wheals induced by the strong histamine solution, compared to those induced by the weak histamine solution and by the saline. Additionally, we observed no increase in skin redness outside the histamine-induced wheals in these healthy dogs. This finding contrasts with observations in humans, where a red area, known as “the flare reaction”, surrounds the histamine wheals.

## 1. Introduction

The intradermal test (IDT) is frequently performed on the lateral thorax of dogs with atopic dermatitis (AD) to identify the environmental allergens to which they are sensitized, with the aim of avoiding exposure and incorporating them into allergen-specific immunotherapy [1]. Unfortunately, the repeatability and reproducibility of IDT results are suboptimal for multiple reasons. These include variation in the volume and depth of intradermal injections; possible differences over lateral thorax in terms of mast cell distribution, the amount of allergen-specific IgE on their surface and releasability, vasculature response to histamine, and the threshold for irritant reactions; administration of injections above or between ribs; and the subjective evaluation of wheal erythema [2]. The latter cause of variability may be minimized using colorimetry [3].

Colorimeters measure the color of the skin surface, and have been used to evaluate the melanin and erythema index of normal canine skin [4], skin color changes after the exposure of Mexican hairless dogs to ultraviolet radiation [5,6], and the skin color of dogs with AD [7,8]. In one study [8], a CL-400^®^ colorimeter connected to a MPA580 Cutometer (Courage-Khazaka, Köln, Germany) showed high intraobserver repeatability for measurement of erythema, when 10 consecutive measurements were taken without removing the probe from the skin surface, and the procedure was repeated at 5 ± 1 min intervals. A disadvantage of this method is that the outer opaque sheath of the probe, which is held in contact with the skin to create a dark chamber, prevents the operator from visually centering the inner color-measuring beam exactly on the desired spot. Therefore, its use for measuring IDT wheal erythema is limited until the amount of probe repositioning required is determined [3]. Also, it is unknown whether erythema is more pronounced and should be measured at the center (where there is probably more vasodilation that will increase erythema, but also more edema that may mask it) [9] or at the peripheral border of the wheals.

When IDT is performed in humans, positive reactions to histamine and allergens are accompanied by erythema (i.e., the flare reaction) expanding well beyond the borders of the wheal [10]. Currently, it is unknown whether a similar phenomenon occurs in dogs and colorimetry may be helpful for investigating this.

The primary aim of this study was to establish the minimum number of colorimetric measurements required to reliably measure erythema at the center and the border of histamine-induced wheals in healthy dogs. A secondary aim was to examine whether intradermal injections of histamine induce a measurable flare reaction 5mm beyond the wheal border.

## 2. Materials and Methods

The study protocol was approved by the Animal Ethics Committee of the Faculty of Veterinary Science, University of Thessaly, Greece (license No 47/24-5-2022). The handling of the dogs complied with the European Communities Council directive 2010/63/EU and state legislation.

A total of 7 healthy dogs, belonging to personnel and students of our faculty, were enrolled. Inclusion criteria required dogs to be at least 6 months old, not pregnant or lactating, with no clinical evidence of cutaneous or systemic disease and no recent administration of systemic or topical medications that could affect the skin’s response to intradermally injected histamine [11]. Written informed consent was obtained from all owners prior to participation in the study.

**Figure 1:**
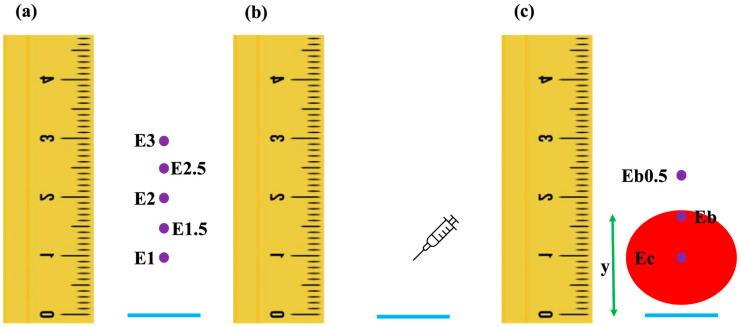
Graphic presentation of the points where skin color was measured before (**a**) and 15 min after (**c**) each intradermal injection (**b**). The horizontal line appears in blue, the purple dots are the points where skin color was measured, the red circle represents the wheal and y is the distance between the horizontal line and the dorsal border of the wheal; (**a**) Three minutes before the injection, skin color was measured at 1 cm, at 1.5 cm, at 2 cm, at 2.5 cm, and at 3 cm dorsally to the horizontal line; (**b**) intradermal injection was performed 1 cm above the horizontal line; (**c**) after 15 min, the distance y was measured, and skin color was measured at the center of the wheal, the border of the wheal, and 0.5 cm dorsally to the border of the wheal.

All procedures were performed in a controlled indoor environment with two windows, adequate artificial illumination and a room temperature between 19 °C and 25 °C [3,12]. The lateral thoracic skin, at approximately equal distance from the spine and sternum, was clipped atraumatically using an electric blade fitted with 1 mm scissors. After 15 min, medetomidine (Domitor^®^, Pfizer-Orion Corporation, Espoo, Finland) was administered intramuscularly at a dose of 20–40 μg/kg. Skin color measurements started once sedation was achieved. For IDT we used the diluent of allergen extracts provided by Nextmune (Lelystad, The Netherlands) as a negative control (NC), and a histamine phosphate solution from the same company at 3 concentrations: the original strength (H1; 0.01%), 10-fold dilution (H2; 0.001%) and 100-fold dilution (H3; 0.0001%); NC was used for both dilutions. All intradermal injections were performed by the same investigator, using 0.5 mL disposable insulin syringes with 30G needles. Each syringe was preloaded with 0.05 mL of solution to ensure consistent injection volume. Injections were disqualified in cases of visible hemorrhage or inadequate wheal formation (i.e., no immediate wheal, wheal of less than the expected size, escape of the solution onto the skin surface). Also, all injections were delivered above the ribs, precisely 1 cm dorsally to a horizontal line (Figure 1), drawn with an atraumatic pen. Each dog received 2 injections of each IDT solution (NC, H1, H2, H3) for a total of eight injections per dog, with injection sites randomly assigned using an online random number generator (https://www.calculator.net/random-number-generator.html, accessed on 10 January 2023). A 3 min interval was allowed between successive intradermal injections.

Exactly 3 min before the first injection, the colorimeter was held perpendicular to the skin surface and the probe was positioned onto the skin with moderate pressure [12]. It was then lifted a few mm above the skin and repositioned, repeatedly, to obtain 30 skin color measurements at each of the following distances dorsal to the horizontal line: 1 cm (E1), 1.5 cm (E1.5), 2 cm (E2), 2.5 cm (E2.5) and 3 cm (E3) (Figure 1a). Following this, the injection of the randomly selected solution (NC, H1, H2 or H3) was performed (Figure 1b), and the same procedure was repeated for the remaining seven injection sites. Exactly 15 min after the first injection, skin color was measured at the center of the wheal (Ec), at the dorsal border of the wheal (Eb) and 0.5 cm dorsally to Eb (Eb0.5). In addition, the distance (y) from the horizontal line to the dorsal border of the wheal was measured (Figure 1c).

The colorimeter CL-400^®^, connected to the MPA580 Cutometer, (Courage-Khazaka, Köln, Germany) was operated by a single investigator who performed all skin color measurements. Recordings that were displayed on a computer screen are based on the CIE L*a*b* system (software MPA CT plus version 1.1.6.9.). Positive values on the a* axis represent measurements of erythema, whereas negative values on the same axis (green spectrum) were discarded [13].

The minimum number (*n*) of repeated measurements required to achieve both normal distribution (Shapiro–Wilk test) in at least 90% and a coefficient of variation (CV) <15% in at least 70% of the 448 datasets [7 dogs × 8 injections/dog × 8 points/injection (5 pre-IDT and 3 post-IDT)] was evaluated; if the analysis showed *n* < 5, then *n* was set at 5. However, these criteria were not met even with 30 measurements (see Results section). Subsequently, the lowest and highest value of the first seven measurements were disqualified and the remaining five measurements were used to create new datasets that were tested for the normality of distribution and for CV. In the subsequent analyses, the mean of the latter datasets (Emean), the disqualified minimum (Emin) and the disqualified maximum (Emax) value of the first seven measurements and the single first measurement (Efirst) of erythema were used.

The change in erythema (*Δ*E) and the percentage change in erythema (%ΔE) at the wheal center (*Δ*Ec and %*Δ*Ec) were calculated by subtracting E1 from Ec (Figure 1). The distributions of *Δ*Ec and %*Δ*Ec were assessed using the Shapiro–Wilk test after pooling the results from all 14 (7 dogs × 2 injections/dog) injection sites for each solution. *Δ*Ec and %*Δ*Ec were compared among NC, H1, H2 and H3 using one-tailed paired samples *t*-tests (normal distribution of differences) or the Wilcoxon matched-pair signed-rank test (non-normal distribution of differences). For solutions where *Δ*Ec and/or %*Δ*Ec differed significantly, and provided that *Δ*Ec and/or %*Δ*Ec were significantly higher for H1 compared to NC, receiver operating characteristic (ROC) curves were generated, and the area under the curve (AUC) along with the cut-off values resulting in the highest Youden index were calculated.

The same analyses were repeated for the change in erythema at the dorsal border of the wheal (*Δ*Eb and %*Δ*Eb) and at a point 0.5 cm dorsally from Eb (*Δ*Eb0.5 and %*Δ*Eb0.5); the latter was performed to examine for a possible flare reaction. *Δ*Eb was calculated by subtracting the pre-IDT measurement of erythema at the site closest to y (i.e., E1.5, E2, E2.5 or E3) from Eb. *Δ*Eb0.5 was calculated by subtracting the pre-IDT measurement of erythema at the site closest to y + 0.5 cm from Eb0.5 (Figure 1).

All analyses were performed using IBM SPSS 29.0.1 for Windows and the level of significance was 5%.

## 3. Results

None of the 56 injections (seven dogs × eight injections/dog) were disqualified due to hemorrhage or injection failure. Due to a logistic error, E1 and Ec (Figure 1) were not measured in two dogs (2 dogs × 8 injections/dog × 2 measuring points × 30 measurements = 960 missing measurements). An additional 1430 measurements were discarded due to negative a* axis values, resulting in a total of 11,050 valid erythema measurements.

Depending on the number of repeated measurements (from 3 to 30), 43.4–92.2% of the datasets were normally distributed; for the predefined minimum *n* (=5) this figure was 87.2%. Also, 43.4–52.7% of datasets had a CV < 15%; for the predefined minimum *n* (=5) this figure was 50.1% (Table 1). Thus, none of the tested numbers of repeated measurements of erythema resulted in a normal distribution or <15% CV in at least 90% and 70% of the datasets, respectively. In contrast, when the lowest and highest values among the first seven measurements were excluded, 93.3% of the resulting datasets showed a normal distribution and 71.8% had CV < 15%. Subsequently Emean, along with Emin, Emax, and Efirst, were further analyzed.

The values of *Δ*E and %*Δ*E at the center of the wheals (*Δ*Ec and %*Δ*Ec) could not be calculated for 19/56 (33.9%) injections due to missing and discarded measurements. Using Emean, *Δ*Ec was significantly higher for H1 compared to NC, H3 and H2, whereas the %*Δ*Ec was significantly higher for H1 compared to NC and H3 but not compared to H2 (Table 2). Emin resulted in no differences in *Δ*Ec or %*Δ*Ec among the four solutions. Emax resulted in significantly higher *Δ*Ec and %*Δ*Ec for H1 compared to H3 and H2 but in no difference between H1 and NC (Table 3). Finally, using Efirst, *Δ*Ec was significantly higher for H1 compared to NC and H3 (but not compared to H2) and significantly higher for H2 compared to H3; also, %*Δ*Ec was significantly higher for H1 compared to H3 and for H2 compared to H3 (Table 4).

The *Δ*E and %*Δ*E at the border of the wheals (*Δ*Eb and %*Δ*Eb) could not be calculated for 13/56 (23.2%) injections due to missing and discarded measurements. The only significant differences were found for Emax, which resulted in significantly higher *Δ*Eb for H1 compared to H3 (AUC: 0.791 at a cut-off of 1.065) and H2 (AUC: 0.785 at a cut-off of 1.03) and in a significantly higher %*Δ*Eb for H1 compared to H3 (AUC: 0.782 at a cut-off of 27.936).

However, 0.5 cm dorsally to the border of the wheals, *Δ*E and %*Δ*E (*Δ*Eb0.5 and %*Δ*Eb0.5) could not be calculated for 5/56 (8.9%) injections due to missing and discarded measurements. The only significant differences were found for Efirst, which resulted in H1 having significantly lower *Δ*Eb0.5 and %*Δ*Eb0.5 values compared to NC (AUC: 0.201 and AUC: 0.205, respectively, both at a cut-off of −0.568).

## 4. Discussion

When this study was designed, it was expected that a reasonable number of repeated measurements obtained by removing and repositioning the colorimeter onto the skin would generate normally distributed datasets with low CV values (i.e., a high precision would be achieved) and, subsequently, that their mean values would be used for the accurate estimation of changes in erythema after IDT. Contrary to our expectations, as the number of consecutive measurements increased, less datasets were normally distributed and the percentage of those with a CV < 15% remained low and showed a tendency to decrease further (Table 1). These findings may be attributed to operator fatigue, resulting in increasing deviation between the desired and the actual skin location where erythema was measured, to the changes in the color of the skin due to the repeated, albeit moderate, pressure applied by the probe [14], and, to the natural change in the degree of erythema during the 3 min measuring window at the sites where histamine solutions had been injected 15–18 min earlier.

For this reason, it was decided to create new datasets by calculating the means of the first seven measurements after excluding the minimum and maximum values, a process analogous to the method recommended for blood pressure measurement in dogs and cats, where the first measurement is discarded and the mean of the subsequent 5–7 measurements is calculated [15]. This resulted in >90% of datasets having normal distribution and >70% of them having a low CV. However, in the absence of a gold standard, it was impossible to determine whether the calculated Emean was representative of the true degree of erythema. For this reason, three additional measures of erythema were analyzed: the Emax (expected to capture the skin spot with the highest degree of erythema after IDT with histamine solutions, but also after IDT with NC, as well as before IDT), the Emin (having the opposite attributes compared to Emax) and Efirst (less chance of probe misposition due to fatigue of the operator). Obtaining repeated measurements of Efirst without removing the probe from the skin was not considered useful for the purposes of this study, because it has already been shown that the repeatability of these measurements is extremely high [8] and, subsequently, their means are not expected to differ substantially from the single first positive a* value.

There was uncertainty about whether it would have been more accurate to measure *Δ*E at the center or at the border of the wheals induced by H1, H2 and H3. At least in theory, the higher histamine concentration at the center [16] was expected to cause maximum vasodilation and thus to result in higher *Δ*E values, but also to maximize vascular permeability and ensuing edema, which may interfere with the measurement of erythema. The results clearly show that *Δ*E should be measured at the center, as none of the four measures of erythema (Emean, Emin, Emax, Efirst) resulted in *Δ*Eb values able to discriminate H1 (expected to have the highest *Δ*E) from NC (expected to have the lowest, if any, *Δ*E) wheals.

Two measures of erythema (Emin and Emax) resulted in a non-significant difference in ΔE at the center between H1 and NC, and thus they are not accurate. Of the remaining two (Emean, Efirst), *Δ*Emean was significantly higher for H1 compared to NC, H3 and H2 and performed better than %*Δ*Emean which could not differentiate H1 from H2. Similarly, %*Δ*Efirst failed to differentiate H1 from NC, but *Δ*Efirst was significantly higher for H1 compared to NC and H3 (but not compared to H2) and was able to differentiate between H3 and H2. In summary, *Δ*Emean and *Δ*Efirst differentiated three out of six wheals that are, theoretically, expected to have different *Δ*E values (NC vs. H3, NC vs. H2, NC vs. H1, H3 vs. H2, H3 vs. H1 and H2 vs. H1). Of the associated AUCs for *Δ*Emean, two out of three were in the acceptable (0.7–0.8) and one out of three in the excellent (0.8–0.9) range, whereas of the *Δ*Efirst AUCs, one of the three was in the acceptable, one in the excellent and one in the outstanding (>0.9) range. Since it is impossible to compare these AUC values statistically, and it is uncertain if finding a significant difference between H1 and H2 but not between H2 and H3 (as was the case with *Δ*Emean) indicates better or worse performance compared to finding significant difference between H2 and H3 but not between H1 and H2 (as was the case with *Δ*Efirst), both measures of erythema are recommended for further studies. These studies should first evaluate whether *Δ*Emean and/or *Δ*Efirst outperform the visual grading of erythema in the differentiation between wheals induced by NC and different histamine dilutions, and, if so, whether they can be used in clinical and/or experimental settings to increase the accuracy of IDT interpretation for environmental allergens.

**Figure 2:**
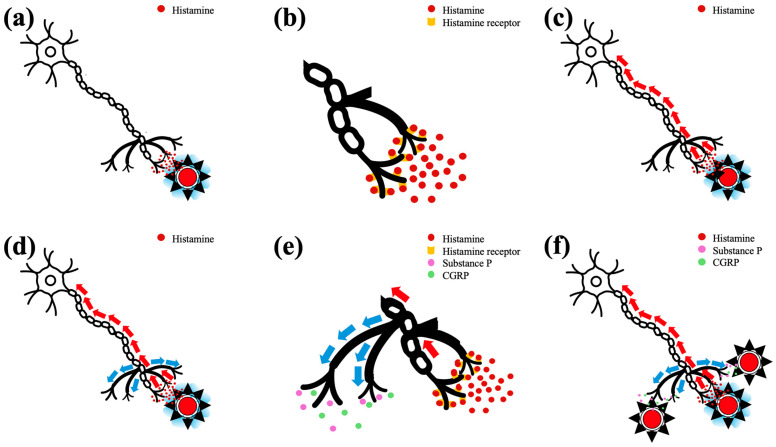
The mechanism of histamine-induced wheal and flare reaction in humans: (**a**) at the site of administration, histamine causes vasodilation and increased vascular permeability leading to wheal formation; (**b**) histamine binds to histamine receptors that are present on local sensory neurons (C-fiber); (**c**) sensory neurons are activated and the impulse is transmitted towards dorsal root ganglia (orthodromic transmission; red arrows); (**d**) the neighboring branches of the sensory nerves are also activated (antidromic transmission; blue arrows); (**e**) the terminals of antidromically activated branches of the sensory neurons release substance P and calcitonin gene-related peptide (CGRP); (**f**) substance P and CGRP cause local vasodilation that is manifested macroscopically as erythema, the so-called “flare reaction”.

In healthy humans, intradermal administration of histamine results in (a) an erythematous wheal at the administration site, caused by the direct effects of histamine on local vasculature (vasodilation and increased vascular permeability), and (b) an erythematous area surrounding the wheal, the so-called “flare reaction” [9]. Flare is not mediated by histamine but by the antidromic activation of neighboring branches of cutaneous C-type sensory nerves that release vasodilatory neuropeptides [9,16,17], namely substance P and calcitonin gene-related peptide (CGRP) [9,17,18] (Figure 2). The amount of histamine needed to bind to histamine receptors, to activate the sensory nerves at the administration site and, finally, to trigger the axon reflex that culminates in the flare reaction, is lower than the amount of histamine needed to induce the wheal [16]. Also, the radius of the wheal is smaller than the radius of the flare [16] and the erythema of flared skin is visually distinct and easily measured by colorimetry [19]. Subsequently, if a similar response to intradermally injected histamine existed in dogs, positive *Δ*E 5 mm dorsally to the border of the wheals, at least those induced by H1, would be expected. Terminals of canine body trunk sensory nerves contain functional histamine receptors [20,21], substance P and CGRP [22]. Therefore, the lack of flare in dogs may be due to the inability of sensory nerves to activate the antidromic stimulation of their neighboring terminals and/or due to lack of substance P and/or CGRP release from antidromically stimulated sensory nerve endings and/or due to a lack of vasodilating properties in these neuropeptides. The latter is more likely, at least for substance P, as its intradermal administration, at pharmacologic doses, in healthy and/or dogs with AD, did not cause visible erythema in two studies [23,24] and resulted in mild erythema in a third [25]. Also, although CGRP at pharmacologic doses causes dilation of canine systemic arteries and veins [26,27], this effect is not uniform among all organs and vascular plexuses [28], and, to the best of our knowledge, has not been examined following intradermal administration of CGRP at pharmacologic doses and, more so, after natural release of this neuropeptide by sensory nerve endings. In any case and irrespective of the exact reason(s) for this difference between humans and dogs, it is more appropriate to use the term “erythematous wheal” rather than “wheal and flare” to describe the response of canine skin to histamine. Also, further studies are needed to investigate whether the lack of flare reaction has clinical, pathophysiological and/or therapeutic implications in canine skin diseases that are mediated by histamine, substance P and/or CGRP, such as urticaria and AD.

The limitations of this study include the relatively small number of dogs along with the large number of missing measurements of erythema, the use of healthy instead of dogs with AD, and the possible influence of medetomidine sedation on the results.

Since this is a proof-of-concept study, it was not possible to perform a priori power calculations. However, despite the small number of dogs and the missing values, as the number of repeated measurements of erythema increased, the percentage of normally distributed datasets gradually decreased (Table 1), and it is highly unlikely that adding more dogs would change this. Also, our data show that Efirst and Emean are superior compared to Emin and Emax for the measurement of *Δ*Ec. Retrospective calculations show that 25 dogs or 13 dogs would be needed to find a significant difference in *Δ*Ec between NC and H1 for Emin and Emax, respectively, whereas this difference was significant for Efirst and Emean using only 7 dogs. However, even if more dogs had been enrolled and more significant differences had been found for Emin and/or Emax, the AUCs would still have been lower compared to Efirst and Emean and, subsequently, the conclusions would have remained the same. As an example, there was a significant difference in *Δ*Emax between H3 and H1, but the AUC was only 0.75 (Table 3), whereas for the same comparison, the AUCs for *Δ*Efirst and *Δ*Emean were 0.903 (Table 4) and 0.861 (Table 2), respectively. Considering the erythema at the border of the wheals, the number of dogs that should have been enrolled to find a significant difference in *Δ*Eb between NC and H1 is 34 for Efirst, 21 for Emin, 19 for Emax, and 140 for Emean. Like before, even if all these dogs had been enrolled, the AUCs would still have been low, as we showed for Emax.

In a single study, the intradermal injection of histamine, at various concentrations, resulted in smaller wheal sizes in dogs with AD compared to healthy dogs [23]. However, no such difference has been found by other investigators [29,30,31]. More importantly, histamine-induced erythema has not been compared between healthy dogs and dogs with AD, and future studies using colorimetry are warranted for this purpose.

Although medetomidine sedation does not affect the subjective score or the size of the wheals that are induced by the intradermal injection of histamine at various concentrations [32], the peripheral vasoconstriction caused by this alpha 2-adrenergic agonist may have reduced the erythema and thus, may have decreased the differences in ΔEc between NC and the three histamine solutions. However, in our study, sedation was necessary to ensure the consistency of the injections in terms of their position (1 cm dorsally to the horizontal line), volume (0.05 mL) and depth.

Further studies are needed to examine if the colorimetric measurement of *Δ*Ec is superior to visual scoring of erythema to differentiate among NC-, H3-, H2- and H1-induced wheals in healthy dogs, and if colorimetry can be used to improve the repeatability and reproducibility of IDT results in dogs with AD.

## 5. Conclusions

Colorimetry can differentiate the change in erythema (*Δ*E) at the center of the wheals (*Δ*Ec) induced in healthy dogs by the intradermal injection of 0.05 mL of 0.01% histamine (H1) from the *Δ*E of the wheals induced by the diluent of allergen extracts (NC) and by 0.0001% histamine (H3). Reliable ways to calculate *Δ*Ec can be achieved by using the first positive a* axis value of the colorimeter before and after the injection or, each time, by obtaining the first seven positive a* axis values, disqualifying the lowest and highest ones and calculating the mean of the remaining five. Unlike in humans, intradermal injection of histamine, even at a concentration of 0.01%, does not induce a flare reaction in healthy dogs.

## Figures and Tables

**Table 1:** Percentage (%) of datasets with normal distribution (NS) and with coefficient of variation (CV) <15% after 3–30 repeated measurements of skin erythema at the same point.

No of Repeated Measurements	% Datasets with NS	% Datasets with CV < 15%
3	92.2%	52.7%
4	88.6%	50.6%
5	87.2%	50.1%
6	84.6%	50.1%
7	83.4%	50%
8	79.8%	49%
9	78.6%	48.3%
10	75.1%	48.4%
11	71.5%	48.2%
12	71.5%	47.7%
13	64.9%	46.5%
14	68%	45.6%
15	66.2%	45.6%
16	65.7%	46.9%
17	61.6%	47.2%
18	61%	48.7%
19	60.2%	45.7%
20	58.7%	45.3%
21	55%	45.8%
22	52.2%	45.5%
23	51.1%	45.2%
24	49.6%	44.5%
25	49%	45.2%
26	48.9%	43.4%
27	47.2%	44%
28	45.6%	44.1%
29	45.7%	44.8%
30	43.4%	45.2%

**Table 2:** Comparisons of the change in mean erythema (*Δ*Emean) and of the percentage change in mean erythema (%*Δ*Emean) at the center of the wheal (Ec), among intradermally injected allergen diluent (NC), histamine 0.0001% (H3), histamine 0.001% (H2) and histamine 0.01% (H1), in 7 healthy dogs. For each comparison with a *p* value < 0.05, the area under the receiver operating characteristic curve (AUC) and the cut-off value resulting in the highest Youden index are presented.

	NC vs. H1	H3 vs. H1	H2 vs. H1
*Δ*Emean			
*p* value	0.028	0.004	0.013
AUC	0.753	0.861	0.733
Cut-off	1.989	−0.024	1.639
%*Δ*Emean			
*p* value	0.034	0.008	0.063
AUC	0.691	0.917	**-**
Cut-off	40.126%	37.341%	**-**

**Table 3:** Comparisons of the change in maximum erythema value (*Δ*Emax) and of the percentage change in maximum erythema value (%*Δ*Emax) at the center of the wheal (Ec), among intradermally injected allergen diluent (NC), histamine 0.0001% (H3), histamine 0.001% (H2) and histamine 0.01% (H1), in 7 healthy dogs. For each comparison with a *p* value < 0.05, the area under the receiver operating characteristic curve (AUC) and the cut-off value resulting in the highest Youden index are presented.

	NC vs. H1	H3 vs. H1	H2 vs. H1
*Δ*Emax			
*p* value	0.053	0.022	0.032
AUC	-	0.750	0.678
Cut-off	-	2.895	2.875
%*Δ*Emax			
*p* value	0.116	0.031	0.016
AUC	**-**	0.708	0.656
Cut-off	**-**	36.758%	26.109%

**Table 4:** Comparisons of the change in the first erythema value (*Δ*Efirst) and of the percentage change in the first erythema value (%*Δ*Efirst) at the center of the wheal (Ec), among intradermally injected allergen diluent (NC), histamine 0.0001% (H3), histamine 0.001% (H2) and histamine 0.01% (H1), in 7 healthy dogs. For each comparison with a *p* value < 0.05, the area under the receiver operating characteristic curve (AUC) and the cut-off value resulting in the highest Youden index are presented.

	NC vs. H1	H3 vs. H2	H3 vs. H1
*Δ*Efirst			
*p* value	0.009	0.014	<0.001
AUC	0.809	0.706	0.903
Cut-off	0.705	1.555	1.555
%*Δ*Efirst			
*p* value	0.088	0.017	0.005
AUC	-	0.738	0.944
Cut-off	-	29.057%	43.6%

## Data Availability

The original contributions presented in this study are included in the article. Further inquiries can be directed to the corresponding author(s).

## Abbreviations

The following abbreviations are used in this manuscript:

AD	Atopic dermatitis
AUC	Area under the curve
CGRP	Calcitonin gene-related peptide
CV	Coefficient of variation
*Δ*E	Change in erythema
*Δ*Eb	Change in erythema at the border of the wheal
*Δ*Eb0.5	Change in erythema 0.5 cm dorsally to the dorsal border of the wheal
*Δ*Ec	Change in erythema at the center of the wheal
*Δ*Efirst	Change in first erythema value
*Δ*Emax	Change in maximum erythema value
*Δ*Emean	Change in mean erythema value
Eb	Erythema at the border of the wheal
Eb0.5	Erythema 0.5 cm dorsally to the dorsal border of the wheal
Ec	Erythema at the center of the wheal
Efirst	First erythema value
Emax	Maximum erythema value
Emean	Mean erythema value
Emin	Minimum erythema value
H1	Histamine 0.01%
H2	Histamine 0.001%
H3	Histamine 0.0001%
IDT	Intradermal test
NC	Negative control
NS	Normal distribution
LD	Linear dichroism
ROC	Receiver operating characteristic
